# Supplementary material for: Genetic Analyses of Mungbean [Vigna radiata (L.) Wilczek] Breeding Traits for Selecting Superior Genotype(s) Using Multivariate and Multi-Traits Indexing Approaches
Source: Plants (Basel). 2023 May 15;12(10):1984. doi: 10.3390/plants12101984 (PMC10223993; doi:10.3390/plants12101984)
Supplement: Supplementary file 1 [file plants-12-01984-s001.zip › plants-2211839-supplementary.pdf]

Table S1. Name, source, and status of the local and exotic mungbean genotypes used in the experiment

Sl. No.	Name of Genotypes/ Cultivars	Status	Gen. Code	Source/Origin/ Place of collection	Sl. No.	Name of Genotypes/ Cultivars	Status	Gen. code	Source/Origin/ Place of collection
1	BMX 94001-3	PRC, Cross	G1	PRC, BARI	61	V01470 VI 000470 AG	Exotic	G61	AVRDC-WVC
2	Samrat	Exotic	G2	IIPR, Kanpur	62	VI 0009 53 AG	Exotic	G62	AVRDC-WVC
3	PM-2	Exotic	G3	IIPR, Kanpur	63	V03063 VI 002063 BG	Exotic	G63	AVRDC-WVC
4	BMXK1 06007	PRC, Cross	G4	PRC, BARI	64	V02579 VI 001579 BG	Exotic	G64	AVRDC-WVC
5	VC 6170(70-92)	Exotic	G5	AVRDC-WVC	65	V02339 VI 001339 AG	Exotic	G65	AVRDC-WVC
6	BMX 97009-2	PRC, Cross	G6	PRC, BARI	66	BARI Mung-6	Released var.	G66	PRC, BARI
7	BMXK1 04005-3	PRC, Cross	G7	PRC, BARI	67	BARI Mung-7	Release var.	G67	PRC, BARI
8	VC 6173 C	Exotic	G8	AVRDC-WVC	68	BARI Mung-3	Release var.	G68	PRC, BARI
9	PM-5	Exotic	G9	IIPR, Kanpur	69	Sukumar	Exotic	G69	IIPR, Kanpur
10	BMXK1 99003-15	PRC, Cross	G10	PRC, BARI	70	IPBM 079-13-29	Exotic	G70	AVRDC-WVC
11	Pusavishal	Exotic	G11	IIPR, Kanpur	71	BU Mung-4	Release var.	G71	BU, Bangladesh
12	VC-6531 B-20	Exotic	G12	AVRDC-WVC	72	V02268 VI 001268 BG	Exotic	G72	AVRDC-WVC
13	PRC-1	PRC, Cross	G13	PRC, BARI	73	BMX 97014-3	PRC, Cross	G73	PRC, BARI
14	BMX 92007-3	PRC, Cross	G14	PRC, BARI	74	VI 004958 BG	Exotic	G74	AVRDC-WVC
15	BMXK1 06006-4	PRC, Cross	G15	PRC, BARI	75	V02579 VI 001579 BG	Exotic	G75	AVRDC-WVC
16	PS-7	Exotic	G16	IARI, New Delhi	76	Mungbean (Chargat)	Local	G76	Chargat
17	BMXK1-06002-2	PRC, Cross	G17	PRC, BARI	77	MB 03	Exotic	G77	SAC, Bangladesh
18	BMXK1 0300011-6	PRC, Cross	G18	PRC, BARI	78	BMX 010015	PRC, Cross	G78	PRC, BARI
19	VC 6170-90	Exotic	G19	AVRDC-WVC	79	BMX 9009-6	PRC, Cross	G79	PRC, BARI
20	BMXK2 0300011-1	PRC, Cross	G20	PRC, BARI	80	MB-07	Exotic	G80	SAC, Bangladesh
21	BMXK1 14004	PRC, Cross	G21	PRC, BARI	81	BMX 007	PRC, Cross	G81	PRC, BARI
22	VC 5153 B-19	Exotic	G22	AVRDC-WVC	82	MB 08	Exotic	G82	SAC, Bangladesh
23	BINA Mung-8	Release var.	G23	BINA	83	Chaingrai Thailand	Exotic	G83	AIT, Thailand
24	BMXK1 07007-4	PRC, Cross	G24	PRC, BARI	84	V06042 VI005041 AG	Exotic	G84	AVRDC-WVC
25	Nilphamari local	Local	G25	Nilphamari Sadar	85	V05957 VI004956 AG	Exotic	G85	AVRDC-WVC
26	BMX 99003-12	PRC, Cross	G26	PRC, BARI	86	V03487 VI002487 AG	Exotic	G86	AVRDC-WVC
27	BMX 07009-10	PRC, Cross	G27	PRC, BARI	87	V05974 VI004973 B-BLM	Exotic	G87	AVRDC-WVC
28	BMX 97024-13	PRC, Cross	G28	PRC, BARI	88	V05932 VI004931 AG	Exotic	G88	AVRDC-WVC
29	BMXK1 0300011-4	PRC, Cross	G29	PRC, BARI	89	V05970 VI004969 AG	Exotic	G89	AVRDC-WVC
30	VC 6173	Exotic	G30	AVRDC-WVC	90	V02557 VI001557 BG	Exotic	G90	AVRDC-WVC
31	BMX 08011-2	PRC, Cross	G31	PRC, BARI	91	V02651 VI001651 BG	Exotic	G91	AVRDC-WVC
32	BMX 09012-2	PRC, Cross	G32	PRC, BARI	92	V01319 VI000319 AG	Exotic	G92	AVRDC-WVC
33	MMB V01	PRC, Cross	G33	RPRS, Madaripur	93	V05872 VI004871 BG	Exotic	G93	AVRDC-WVC
34	BMXK2 04005-3	PRC, Cross	G34	PRC, BARI	94	V05935 VI004934 AG	Exotic	G94	AVRDC-WVC
35	BMX 01019-1	PRC, Cross	G35	PRC, BARI	95	V01253 VI000253 AG	Exotic	G95	AVRDC-WVC
36	SMZ 134	Exotic	G36	ACIAR, Australia	96	V04364 VI003364 AG	Exotic	G96	AVRDC-WVC
37	BINA Mung-7	Release var.	G37	BINA,	97	V02268 VI001268 BG	Exotic	G97	AVRDC-WVC
38	GK-22	Exotic	G38	ACIAR, Australia	98	V02974 VI001974 BG	Exotic	G98	AVRDC-WVC
39	BMX 94001-3	PRC, Cross	G39	PRC, BARI	99	V03569 VI002569 BG	Exotic	G99	AVRDC-WVC
40	MMV12	PRC, Cross	G40	RPRS, Madaripur	100	V04251 VI003251 A-BL	Exotic	G100	AVRDC-WVC
41	BMX 05009	PRC, Cross	G41	PRC, BARI	101	V04928 VI003927 AG	Exotic	G101	AVRDC-WVC
42	BMX 97014-3	PRC, Cross	G42	PRC, BARI	102	V02579 VI001579 BG	Exotic	G102	AVRDC-WVC
43	MMAT V07	PRC, Cross	G43	RPRS, Madaripur	103	V02692 VI001692 AG	Exotic	G103	AVRDC-WVC
44	BMX 050012-6	PRC, Cross	G44	PRC, BARI	104	V03432 VI002432 AG	Exotic	G104	AVRDC-WVC
45	BINA Mung-6	Release var.	G45	BINA,	105	V04952 VI003951 AG	Exotic	G105	AVRDC-WVC
46	SMZ-37	Exotic	G46	ACIAR, Australia	106	V02698 VI001698 BG	Exotic	G106	AVRDC-WVC
47	BMXK1 08011-8	PRC, Cross	G47	PRC, BARI	107	V03051 VI002051 BG	Exotic	G107	AVRDC-WVC
48	Thailand	Exotic	G48	ChaingMai	108	V01766 VI000766 BG	Exotic	G108	AVRDC-WVC
49	BMXK2 0300	PRC, Cross	G49	PRC, BARI	109	V05876 VI004877 AG	Exotic	G109	AVRDC-WVC
50	BMXK1 07007-10	PRC, Cross	G50	PRC, BARI	110	V04735 VI003734 B-DG	Exotic	G110	AVRDC-WVC
51	VI 004954 BG	Exotic	G51	AVRDC-WVC	111	IPBM-79-13-29	Exotic	G111	ACIAR
52	BMXK1 07007-4	PRC, Cross	G52	PRC, BARI	112	Sonamung (Balidanga)	Local	G112	Thakurgaon
53	V04952 VI 003951 AG	Exotic	G53	AVRDC-WVC	113	Sonali (PRC-1)	Local	G113	PRC, BARI
54	V02221 VI0011221	Exotic	G54	AVRDC-WVC	114	880032-3	Local	G114	ACIAR
55	V03739 VI 002739	Exotic	G55	AVRDC-WVC	115	Sonamung (Kaligonj)	Local	G115	Jhenaidha
56	VC 6170-92	Exotic	G56	AVRDC-WVC	116	84-2-24	Local	G116	ACIAR
57	VI 002432 AG	Exotic	G57	AVRDC-WVC	117	VO1175-VI000175BY	Exotic	G117	AVRDC-WVC
58	V03647 VI 002647	Exotic	G58	AVRDC-WVC	118	Sonamung	Local	G118	PRC, BARI
59	Jiangsu-1 China	Exotic	G59	Nanjin, China	119	Sonamung (Methapukur)	Local	G119	Rangpur
60	Jiangsu-3 China	Exotic	G60	Nanjin, China	120	189	Local	G120	ACIAR

Table S1 Continued

Sl.. No.	Genotypes/Cultivars	Status	Gen. Code	Source/Origin/ Place of collection	Sl.. No.	Genotypes/Cultivars	Status	Gen. Code	Source/Origin/ Place of collection
121	Sonamung, Bhangga	Local	G121	Bhangga, Faridpur	144	Sonamung, Faridpur	Local	G144	Faridpur sadar
122	6017	Local	G122	ACIAR, Australia	145	V06023 VI 005022 BG	Exotic	G145	AVRDC-WVC
123	Sonamung, Shibgonj,	Local	G123	Shibgonj, Nawabgonj	146	VO 1175 VI 00175 BY	Exotic	G146	AVRDC-WVC
124	BMX870006-9	PRC, Cross	G124	PRC, BARI	147	VO 4561 VI 003560 BG	Exotic	G147	AVRDC-WVC
125	Sonamung, Sadarpur	Local	G125	Sadarpur, Faridpur	148	Sonamung, Safigonj	Local	G148	Safigonj, Noakhali
126	BMX9009-7	Exotic	G126	ACIAR, Australia	149	Sonamung, Char Shularia	Local	G149	Noakhali
127	PAGARA-2	Exotic	G127	ACIAR, Australia	150	Sonamung, Khalishtola	Local	G150	Noakhali
128	F-8-1	Exotic	G128	ACIAR, Australia	151	Sonamung, Char Jabbar	Local	G151	Noakhali
129	Sonamung, Tongipara	Local	G129	Tongipara, Gopalgonj	152	Sonamung, Sariakandi	Local	G152	Bogura
130	BMX880036-5	PRC, Cross	G130	PRC, BARI	153	BARI Mung-8	Release Var.	G153	PRC, BARI
131	Thakurgaon local	Local	G131	ARS, Thakurgaon	154	Sonamung, Raj	Local	G154	Court, Rajshahi
132	BMLX900010-26	PRC, Cross	G132	PRC, BARI	155	Thakurgaon local	Local	G155	ARS, Thakurgaon
133	Sonamung, Chakbazar	Local	G133	Chakbazar, Faridpur	156	Dinajpur local	Local	G156	Dosmile, Dinajpur
134	870006-9	Exotic	G134	PRC, BARI	157	V0 20 VI 00020 AY	Exotic	G157	AVRDC-WVC
135	V0120 V100020	Exotic	G135	AVRDC-WVC	158	Char Torabali (Noakhali)	Local	G158	Noakhali
136	8800115	Exotic	G136	ACIAR, Australia	159	Rhonpur (Chapai)	Local	G159	Chapainawbganj
137	BML-84-2-24	Exotic	G137	ACIAR, Australia	160	Charlaximipur (Faridpur)	Local	G160	Faridpur
138	Sonamung, Tepakhola,	Local	G138	Tepakhola, Faridpur	161	Jhenaidha local	Local	G161	Sadar, Jhenaidha
139	A-47-4	Exotic	G139	ACIAR, Australia	162	Sonali (PRC-3)	Local	G162	PRC, BARI
140	Sonamung	Local	G140	Goalchamot, Faridpur	163	Sonali (PRC-4)	Local	G163	PRC, BARI
141	Sonamung, Godagari	Local	G141	Godagari, Rajshahi	164	Chuadanga local	Local	G164	Sadar, Chuadanga
142	Sonali (PRC-2)	Local	G142	PRC, BARI	165	Netrocona local	Local	G165	Sadar, Netrocona
143	Sonamung, Jalalabad	Local	G143	Jalalabad, Gopalgonj	166	Pirojpur local	Local	G166	Sadar, Pirojpur

Released Var. –Released Variety

Table S2. Physical and chemical properties of soil in the experimental with the extraction technique

Properties	Values (%)	Extraction technique
**Physical soil properties**		
Sand	61	-
Silt	23	-
Clay	16	-
Textural class	Sandy loam-clay loam	Hydrometer method [75]. Determined by Marshall's triangular coordinates by USDA system
**Chemical soil parameters**		
Organic matter (%)	1.25	Wet oxidation method [75]. Calculated by Van Bemmelen factor 1.73 [76]
pH	7.6	Glass-electrode pH meter with 1:1.25 soil-water ratios [77]
Total N (g/kg)	0.065	Micro-Kjeldahl method [78]
Available P (ppm)	12.01	Molybdate blue ascorbic acid [78].
Exchangeable K (meq 100 g/soil)	0.16	Determined by flame photometer [78]
Available S (ppm)	10.12	Turbidity method using $BaCl_2$ [79]
Available B (ppm)	1.20	Calcium chloride extraction method [77]
Available Zn (ppm)	0.19	Atomic absorption spectrophotometer [80]

Table S3. Studied qualitative traits, assigned scores based on descriptors, and evaluation stage for mungbean genotypes

Sl. No.	Qualitative traits	Descriptor states and their scores	Evaluation stage
1	Hypocotyl colour	1- Green, 2- Green-purple, 3- Purple, 4- Dark purple, 5- Mixed and 6-Others	At 10 days after emergence
2	Seedling vigour	3- Poor, 5- Intermediate, and 7- Vigorous	At 15 days after emergence
3	Stem colour	1- light green, 2- dark green, 3- light purple, 4- dark purple, and 5- others	At 50% flowering
4	Stem pubescence	1- Glabrous, 2- Sparsely pubescent, 3- Moderately pubescent, and 4- Highly pubescent	At 50% flowering
5	Leaf pubescence	1- Glabrous, 2- Very sparsely, 3- Sparsely pubescent, 4- Moderately pubescent, and 5- Densely pubescent	At 50% flowering
6	Leaf colour	3- Light green, 5- Intermediate green, and 7- Dark green	At 50% flowering)
7	Leafiness	1- Sparse, 2- Intermediate, and 3- Abundant	At 50% flowering
8	Terminal leaflet shape	1- Deltate, 2- Ovate, 3- Ovate-lanceolate, 4 - Lanceolate and 5- Rhombic	At 50% flowering
9	Calyx colour	1- Green, 2- Purplish green, 3- Greenish purple, and 4- Others	At 50% flowering
10	Corolla colour	1- Yellow, 2- Greenish yellow, 3- Yellowish green, 4-. Green-purplish yellow and 5-Others	At 50% flowering
11	Pod beak shape	1- Pointed, 2- Blunt (Round), and 3- Others	At maturity
12	Mature pod colour	1- Straw, 2- coffee (chocolate), 3- Brown, 4- Brown and black, 5- Black and 6-Others	At maturity
13	Pod curvature	1- Straight, 2- Slightly curved, and 3- Curved (sickle-shaped)	At maturity
14	Seed colour	1- Light green, 2- Dark green, 3-Light yellow, 4- Yellow, 5-Brown and 6-Mottled	After harvest
15	Seed stature	1- Small, 2- Medium and 3-Bold	After harvest

Table S4. Quantitative traits, abbreviations, measurement procedure, and evaluation phase of mungbean genotypes

Sl. No.	Quantitative traits	Abbreviations	Measurement procedure	Evaluation phase
1	Days to first flowring	DFF	The number of days taken from sowing to the appearance of the first flower in the population of each genotype.	Growth stage
2	Days to flowering (50%)	DF	The number of days to flowering was quantified from the planting date to the day when 50% flowering was in the population of each genotype.	At 50% flowering
3	Days to maturity	DM	The number of days taken from sowing to stage when 80% of the plant has mature pods.	At maturity
4	Plant height (cm)	PH	Plant height was measured from the ground to the tip of the main shoot of 5 randomly selected plants on a meter scale at the time of harvest.	At maturity
5	Canopy temperature	CT	Canopy temperature was measured at 50% flowering stage by the infrared thermometer (Model THI-500, TASCO, Japan) on the day during 12:00-02:00 pm.	At 50% flowering
6	SPAD value	SV	The SPAD chlorophyll meter determines the leaf greenness which is a direct measure of leaf chlorophyll content and an indirect method of leaf nitrogen. The SPAD chlorophyll meter readings were taken 40 days after sowing.	At 50% flowering
7	Number of pods per plant	NPPP	The total number of pods per plant was calculated by the summation of pods in five selected pants at the harvesting stage and the average value of the data was recorded as the number of pods per plant.	At maturity
8	Number of pods per cluster	NPPC	The numbers of pods per cluster were calculated at the maturity stage from five randomly selected plants and the value averaged over per cluster was counted.	At maturity
9	Number of seeds per pod	NSPP	The total number of seeds per pod was counted from five maturity pods collected from five randomly selected plants. The pods were collected at the mature stage and average seeds per pod were calculated.	After harvest
10	pod weight with seed	PWtS	An average of 10 pods was collected from 5 randomly selected plants. The pods were collected at the mature stage, cleaned, and weighed to get the pod weight in grams. The average weight was calculated.	After harvest
11	Shell weight per pod	ShWtPP	Dry shell weight of randomly selected 10 pods from five selected plants was taken after removing seeds at dry condition. Finally, per pod basis weight was calculated.	After harvest
12	Seed weight per pod	SWtPP	The weight of seeds of ten randomly taken pods was measured in grams after moisture was adjusted to 10-12% and then divided by ten. Finally, per pod basis weight was measured.	After harvest
13	Pod length	PL	Exterior distance of fully matured pod from the pod apex to the peduncle measured in centimeters at physiological maturity from an average of mature 10 pods taken at random.	After harvest
14	100-seed weight	HSW	One thousand dried seeds from 5 randomly selected in each genotype were counted and weighted by the digital balance at 10-12% moisture level. The weighted values were expressed in grams.	After harvest
15	Yellow mosaic virus	YMV	The severity of yellow mosaic virus infection in mungbean was collected from each replication in each genotype at 45 and 55 days after sowing (DAS).	Vegetative
16	Yield per plant	YPP	Dried grain yield in grams, obtained from 10 plants within central rows of each plot was harvested and threshed separately. The cleaned seeds were weighed in grams at the moisture level is 10-12% and then divided by ten.	After harvest
17	Stover yield per plant	SYPP	Stover yield was taken from 5 randomly selected plants on an oven dry weight basis after harvest. The plants' samples were dried at 70-degree Celsius for 72 hours in an oven. Average stover weight was recorded using electric balance and then per plant basis weight was calculated.	After harvest
18	Biological yield per plant	BYPP	An average of 5 plants were uprooted, cleaned, and weighted to get the biological yield per plant in grams.	Calculated
19	Harvest index	HI	Harvest index (HI) was determined as stover yield expressed as %age of total biological yield HI = (SY/BY) × 100.	Calculated

Table S5. Mean data of studied quantitative yield associated traits of mungbean genotypes in Season 1 (2018)

Genotypes	DFF		DF		DM		PH		CT		SV		NPPP		PPC		SPP		SPWt		ShWtPP		SWtPP	
	Mean	± S.E.	Mean	± S.E.	Mean	± S.E.	Mean	± S.E.	Mean	± S.E.	Mean	± S.E.	Mean	± S.E.	Mean	± S.E.	Mean	± S.E.	Mean	± S.E.	Mean	± S.E.	Mean	± S.E.
G1	35.50	0.50	40.17	0.17	61.83	0.60	64.31	0.14	27.68	0.02	39.15	0.37	18.64	0.16	4.18	0.29	13.39	0.45	0.79	0.02	0.48	0.03	0.31	0.01
G2	35.33	0.44	40.00	0.76	60.83	0.44	68.11	0.35	27.87	0.25	32.80	0.14	24.39	0.76	4.28	0.42	11.63	0.45	0.73	0.02	0.36	0.02	0.37	0.02
G3	36.17	1.64	40.83	1.42	58.00	0.50	64.86	0.28	28.70	0.01	43.14	0.77	38.28	0.85	4.96	0.18	11.59	0.74	0.74	0.04	0.33	0.04	0.41	0.00
G4	37.67	0.88	42.33	0.88	59.83	0.60	64.49	0.28	28.74	0.23	43.38	0.09	25.94	0.63	4.34	0.29	14.91	0.32	1.28	0.04	0.61	0.03	0.66	0.03
G5	35.83	0.44	40.50	0.29	59.83	0.67	64.51	0.31	27.54	0.23	38.11	0.18	26.08	0.79	5.64	0.09	9.99	1.00	0.97	0.01	0.47	0.07	0.51	0.06
G6	33.50	1.04	38.17	0.73	58.50	1.04	73.50	0.25	28.15	0.18	27.56	0.19	35.11	0.44	5.03	0.32	13.21	1.27	0.86	0.01	0.35	0.04	0.51	0.04
G7	32.17	0.73	36.83	0.83	59.33	0.44	52.20	0.55	24.91	0.15	39.15	0.15	28.15	0.79	3.17	0.04	13.56	0.87	1.02	0.05	0.28	0.08	0.74	0.04
G8	34.67	0.60	39.33	0.93	56.83	0.44	63.52	0.20	23.16	0.32	43.83	0.43	23.62	0.72	4.02	0.27	11.52	0.44	0.88	0.04	0.31	0.08	0.58	0.04
G9	36.67	0.44	41.33	0.17	58.50	0.58	67.76	0.48	24.15	0.13	33.85	0.33	27.10	0.90	5.66	0.49	11.90	0.50	0.95	0.01	0.37	0.01	0.58	0.02
G10	32.83	0.17	37.50	0.29	56.17	0.67	88.37	0.51	24.60	0.23	33.85	0.33	15.34	0.81	4.10	0.32	11.86	0.75	0.74	0.04	0.28	0.06	0.47	0.03
G11	31.17	0.83	35.83	1.17	60.00	0.29	71.93	0.30	28.53	0.07	42.74	0.27	13.17	0.60	4.49	0.64	12.37	0.83	0.92	0.03	0.23	0.04	0.69	0.05
G12	33.00	0.50	37.67	0.44	62.00	0.76	63.09	0.30	23.98	0.15	43.98	0.31	34.97	0.38	4.34	0.16	12.71	0.62	1.30	0.04	0.50	0.03	0.80	0.04
G13	35.83	0.88	40.50	0.58	59.67	0.17	74.36	0.20	24.74	0.14	41.03	0.25	23.58	0.76	4.17	0.15	11.49	0.32	1.04	0.01	0.45	0.02	0.59	0.01
G14	35.50	0.50	40.17	0.44	62.67	1.30	58.34	0.37	22.72	0.24	43.37	0.14	16.43	0.48	4.43	0.24	11.18	1.00	0.88	0.01	0.48	0.03	0.40	0.03
G15	32.50	0.50	37.17	0.73	62.17	0.33	64.73	0.26	24.37	0.30	43.40	0.08	10.74	0.86	3.75	0.58	10.88	0.39	0.75	0.03	0.34	0.03	0.41	0.01
G16	35.67	0.73	40.33	1.01	60.00	0.76	68.09	0.14	18.31	0.32	33.41	0.40	15.98	0.69	3.83	0.62	11.69	0.26	0.97	0.01	0.56	0.03	0.41	0.02
G17	31.83	0.44	36.50	0.76	58.17	0.60	59.12	0.35	17.43	0.20	34.33	0.24	22.00	0.89	4.34	0.77	12.33	0.25	1.04	0.01	0.38	0.02	0.67	0.03
G18	35.33	0.33	40.00	0.58	57.33	0.83	68.05	0.36	18.68	0.09	28.08	0.23	20.59	0.72	4.50	0.09	11.70	1.04	1.05	0.02	0.59	0.04	0.47	0.02
G19	31.67	0.17	36.33	0.44	57.33	0.44	73.89	0.29	17.91	0.31	43.31	0.47	16.93	0.70	3.38	0.22	12.71	0.62	1.28	0.04	0.60	0.02	0.68	0.05
G20	33.83	0.88	38.50	0.58	59.83	0.17	63.16	0.29	18.33	0.05	29.16	0.42	19.78	0.43	5.29	0.51	13.38	0.70	0.97	0.01	0.25	0.04	0.72	0.05
G21	35.33	0.73	40.00	0.50	60.00	0.50	57.92	0.43	17.47	0.33	36.96	0.23	10.73	0.64	4.17	0.49	11.87	0.75	1.07	0.01	0.51	0.03	0.56	0.02
G22	34.00	1.50	38.67	1.17	62.00	0.50	65.04	0.25	17.44	0.28	37.96	0.30	25.47	0.26	3.71	0.24	12.98	0.75	1.00	0.03	0.41	0.03	0.59	0.01
G23	33.00	0.76	37.67	0.44	59.83	0.17	67.72	0.23	22.91	0.19	37.61	0.42	15.97	0.19	4.50	0.09	12.91	0.69	1.00	0.02	0.41	0.02	0.58	0.00
G24	32.00	0.29	36.67	0.17	58.83	0.67	58.47	0.29	17.72	0.09	49.13	0.43	20.06	0.45	4.40	0.40	12.64	0.31	1.01	0.02	0.50	0.03	0.51	0.01
G25	36.83	0.88	41.50	0.58	58.83	0.44	63.49	0.22	17.62	0.18	36.89	0.40	19.14	0.84	3.91	0.43	11.69	0.04	0.63	0.05	0.34	0.04	0.29	0.02
G26	32.83	0.33	37.50	0.00	61.50	0.29	53.28	0.22	14.81	0.14	33.05	0.48	18.83	0.72	4.29	0.28	12.36	0.99	0.90	0.01	0.45	0.02	0.45	0.02
G27	33.67	0.93	38.33	0.60	58.50	0.50	53.94	0.28	12.41	0.10	33.61	0.09	19.78	0.73	3.90	0.27	12.54	0.36	1.12	0.05	0.55	0.07	0.57	0.02
G28	32.00	0.58	36.67	0.67	62.17	0.44	53.95	0.21	12.46	0.23	34.12	0.35	15.66	0.50	4.18	0.31	13.05	0.66	1.07	0.01	0.42	0.03	0.65	0.04
G29	31.00	0.29	35.67	0.60	63.33	0.33	68.56	0.24	12.75	0.34	36.89	0.29	23.58	0.76	3.50	0.34	11.56	0.91	0.99	0.03	0.38	0.03	0.62	0.01
G30	32.50	0.29	37.17	0.17	60.67	0.73	63.61	0.40	12.22	0.30	34.12	0.35	16.26	0.41	4.90	0.62	13.15	0.46	1.00	0.03	0.54	0.03	0.45	0.02
G31	36.67	0.44	41.33	0.17	59.00	0.29	73.47	0.33	13.52	0.51	37.16	0.19	14.39	0.80	4.59	0.44	13.46	0.48	0.77	0.01	0.20	0.02	0.57	0.03
G32	32.33	0.33	37.00	0.58	60.83	0.60	63.23	0.50	13.08	0.14	43.08	0.33	10.59	0.77	3.61	0.43	12.74	0.52	1.01	0.02	0.33	0.03	0.68	0.01
G33	37.50	0.29	42.17	0.44	64.00	0.50	73.63	0.30	12.25	0.18	43.39	0.40	13.44	0.79	4.28	0.43	12.03	0.57	0.89	0.03	0.46	0.01	0.44	0.01
G34	35.17	0.73	39.83	0.44	59.50	0.58	59.37	0.39	12.45	0.20	38.91	0.07	10.91	0.99	5.09	0.33	12.20	0.85	1.00	0.03	0.44	0.04	0.56	0.03
G35	35.17	0.33	39.83	0.33	60.00	1.00	58.97	0.54	12.99	0.11	38.54	0.16	15.03	0.68	3.87	0.18	11.70	0.80	1.14	0.06	0.43	0.08	0.71	0.02
G36	35.00	1.44	39.67	1.17	60.83	0.93	68.53	0.25	13.14	0.08	38.54	0.16	15.49	0.62	4.62	0.40	12.88	0.19	0.72	0.02	0.29	0.04	0.43	0.02
G37	35.33	0.44	40.00	0.50	59.00	0.29	58.64	0.20	12.87	0.30	39.09	0.21	17.84	0.20	5.32	0.14	12.85	0.22	1.00	0.03	0.34	0.03	0.66	0.02
G38	31.83	0.44	36.50	0.50	60.50	0.58	53.47	0.12	14.73	1.44	27.04	0.36	27.80	0.75	4.09	0.17	10.44	0.18	1.08	0.02	0.54	0.02	0.54	0.03
G39	35.00	0.29	39.67	0.60	62.33	1.48	59.70	0.15	13.25	0.19	39.07	0.12	12.49	0.07	4.44	0.17	12.88	0.32	0.93	0.04	0.26	0.03	0.67	0.04
G40	36.50	0.50	41.17	0.44	61.33	0.17	67.86	0.31	13.44	0.24	38.72	0.36	17.56	0.32	3.63	0.06	11.86	0.75	0.78	0.02	0.38	0.02	0.40	0.02
G41	39.00	0.29	43.67	0.44	60.83	0.67	63.88	0.15	17.98	0.30	39.02	0.52	9.15	0.28	3.93	0.16	11.69	0.64	0.89	0.02	0.43	0.03	0.47	0.01
G42	38.33	0.88	43.00	1.00	63.33	0.33	64.07	0.38	23.45	0.14	38.89	0.52	11.54	0.56	4.32	0.28	12.87	1.14	1.01	0.03	0.26	0.02	0.75	0.04
G43	37.00	0.29	41.67	0.17	60.50	0.29	59.82	0.14	20.40	0.27	36.82	0.12	28.15	0.40	3.66	0.14	12.71	0.62	0.78	0.03	0.33	0.01	0.44	0.02
G44	36.50	1.04	41.17	0.83	62.00	0.50	78.42	0.40	18.49	0.24	43.79	0.43	22.48	0.37	4.13	0.15	12.27	0.50	1.06	0.00	0.41	0.04	0.65	0.04
G45	37.33	0.44	42.00	0.76	62.00	0.58	73.88	0.47	20.00	0.67	44.07	0.40	29.56	0.51	4.70	0.14	11.79	0.29	0.88	0.03	0.44	0.03	0.44	0.01
G46	37.50	0.29	42.17	0.44	58.67	0.17	63.91	0.38	18.63	0.37	33.78	0.36	39.97	1.03	7.05	0.21	11.86	0.60	0.88	0.03	0.26	0.05	0.62	0.02
G47	37.50	0.29	42.17	0.17	59.17	0.88	88.12	0.43	19.65	0.24	31.09	0.18	20.69	1.20	5.07	0.25	11.29	0.74	0.90	0.02	0.33	0.01	0.57	0.02
G48	35.83	0.60	40.50	0.29	63.00	0.29	58.81	0.24	20.29	0.29	31.07	0.07	14.36	0.84	3.64	0.13	11.75	0.15	0.89	0.03	0.24	0.05	0.65	0.03
G49	37.67	0.67	42.33	0.88	60.33	0.44	68.07	0.28	20.50	0.26	32.82	0.13	32.77	1.14	5.13	0.22	12.04	0.77	1.00	0.02	0.39	0.05	0.61	0.03
G50	33.67	0.60	38.33	0.60	57.83	0.17	63.79	0.04	21.51	0.52	38.18	0.40	17.24	0.82	5.54	0.24	12.82	1.17	0.98	0.03	0.31	0.04	0.67	0.01
G51	32.00	0.29	36.67	0.17	59.67	0.17	64.52	0.04	19.82	0.35	37.29	0.11	11.54	0.78	4.28	0.26	12.00	0.48	0.77	0.03	0.37	0.02	0.41	0.03
G52	39.50	0.58	44.17	0.88	60.33	0.17	67.74	0.26	19.40	0.37	34.22	0.16	18.83	0.72	4.26	0.17	12.71	0.04	0.90	0.02	0.45	0.04	0.45	0.02
G53	32.00	1.04	36.67	1.17	60.00	0.50	59.34	0.27	17.64	0.60	44.22	0.47	10.59	0.77	3.59	0.10	11.58	0.85	0.89	0.03	0.47	0.01	0.42	0.02
G54	32.67	0.73	37.33	0.44	60.33	1.36	68.57	0.51	17.99	0.35	37.53	0.33	7.71	0.79	4.78	0.15	11.01	0.16	0.76	0.04	0.35	0.07	0.41	0.03
G55	37.00	0.76	41.67	0.60	61.17	0.93	74.81	0.43	18.09	0.29	28.10	0.24	24.49	0.98	6.18	0.15	10.98	0.60	0.89	0.03	0.32	0.05	0.57	0.03
G56	33.67	1.09	38.33	0.93	58.33	0.60	59.83	0.37	18.71	0.25	43.88	0.31	41.80	0.76	3.82	0.33	11.70	0.64	0.98	0.02	0.42	0.02	0.57	0.02
G57	41.33	0.44	46.00	0.76	65.50	1.00	51.75	0.65	19.86	0.11	43.25	0.44	9.49	0.10	3.98	0.23	10.78	0.18	0.87	0.07	0.19	0.03	0.68	0.05
G58	38.00	0.00	42.67	0.33	65.67	0.73	52.65	0.18	14.34	0.11	44.00	0.55	7.10	0.18	4.57	0.33	10.33	0.60	0.98	0.03	0.34	0.03	0.64	0.01
G59	36.17	0.67	40.83	0.33	58.50	0.00	68.94	0.46	13.07	0.07	37.70	0.18	21.85	0.58	4.17	0.27	12.44	0.58	0.83	0.04	0.29	0.04	0.53	0.04
G60	36.33	0.73	41.00	0.76	60.17	0.67	74.43	0.56	17.07	0.42	46.51	0.06	11.40	0.49	4.20	0.29	11.25	1.18	1.00	0.03	0.29	0.05	0.71	0.04
G61	33.00	0.29	37.67	0.17	60.33	0.83	77.56	0.18	13.84	0.26	47.99	0.11	16.31	0.47	4.05	0.30	12.10	1.31	0.99	0.02	0.33	0.04	0.65	0.03
G62	35.00	0.50	39.67	0.73	65.67	1.01	77.84	0.25	18.00	0.12	59.97	0.42	9.81	0.22	3.85	0.21	11.67	1.33	0.82	0.04	0.37	0.02	0.45	0.03
G63	31.00	0.50	35.67	0.83	66.00	0.50	69.11	0.78	13.05	0.07	26.08	0.39	5.20	0.18	4.54	0.43	12.16	0.26	0.82	0.04	0.22	0.07	0.60	0.03
G64	34.83	0.93	39.50	0.87	61.00	0.50	48.63	0.54	12.99	0.39	37.45	0.29	9.33	0.34	4.42	0.32	10.93	0.72	0.68	0.03	0.28	0.03	0.40	0.03
G65	33.67	1.42	38.33	1.64	64.67	1.20	68.31	0.46	13.33	0.07	19.96	0.27	12.04	0.84	4.18	0.42	11.81	0.50	1.32	0.05	0.68	0.04	0.65	0.02
G66	33.67	0.60	38.33	0.93	58.00	0.50	64.27	0.19	12.54	0.30	44.11	0.42	20.90	0.57	4.51	0.45	11.39	0.72	0.80	0.01	0.27	0.02	0.53	0.03
G67	33.67	0.93	38.33	0.60	57.50	0.58	59.03	0.07	13.08	0.12	43.70	0.33	18.36	0.37	3.78	0.23	12.31	0.50	0.85	0.03	0.19	0.01	0.66	0.01
G68	34.00	0.00	38.67	0.33	60.50	0.29	58.37	0.54	13.32	0.28	33.77	0.37	12.81	0.48	4.23	0.48	11.83	0.62	0.64	0.04	0.23	0.02	0.42	0.02
G69	33.83	0.60	38.50	0.29	58.83	0.44	64.71	1.46	13.24	0.20	33.30	0.64	31.17	0.30	4.58	0.62	11.08	0.47	0.52	0.02	0.25	0.04	0.27	0.02
G70	32.83	0.67	37.50	0.58	62.50	0.58	49.12	1.47	14.19	0.18	37.10	0.17	12.03	0.30	4.09	0.19	11.18	0.59	1.04	0.03	0.32	0.05	0.71	0.05
G71	34.33	1.17	39.00	0.87	61.00	1.04	86.97	0.19	12.94	0.11	43.43	0.18	13.44	0.38	4.86	0.13	12.82	0.86	1.10	0.04	0.53	0.05	0.57	0.02
G72	32.83	0.60	37.50	0.87	61.50	0.58	57.15	0.93	13.61	0.34	33.68	0.42	19.13	0.12	3.92	0.16	11.42	0.77	0.83	0.04	0.29	0.04	0.54	0.03
G73	34.17	0.83	38.83	1.17	58.33	0.60	64.59	0.66	13.15	0.37	36.80	0.10	10.27	0.16	3.60	0.29	11.87	0.88	1.06	0.02	0.50	0.04	0.56	0.03
G74	35.00	1.00	39.67	1.33	61.83	0.33	74.48	0.20	12.73	0.34	38.97	0.28	15.83	0.33	3.81	0.50	12.20	0.33	0.72	0.02	0.30	0.03	0.42	0.02
G75	32.33	0.17	37.00	0.29	58.83	0.67	53.39	0.65	13.56	0.25	37.77	0.34	10.44	0.09	5.00	0.13	12.03	1.00	1.25	0.04	0.75	0.04	0.50	0.02
G76	34.50	0.76	39.17	0.93	59.67	0.44	49.56	0.31	13.26	0.35	34.21	0.39	10.40	0.08	4.73	0.06	11.86	0.32	0.66	0.04	0.26	0.04	0.41	0.01
G77	33.33	0.60	38.00	0.76	57.00	1.80	52.92	0.22	14.32	0.14	43.81	0.42	13.93	0.31	4.35	0.21	11.08	0.53	0.93	0.04	0.38	0.05	0.55	0.02
G78	34.83	1.09	39.50	0.76	61.33	0.17	55.60	0.36	16.94	0.32	33.98	0.31	18.05	0.59	5.94	0.72	11.02	0.72	0.93	0.01	0.31	0.02	0.63	0.02
G79	33.17	0.17	37.83	0.17	59.33	0.73	59.72	0.39	17.76	0.27	42.85	0.47	27.23	0.41	5.09	0.16	11.42	0.82	0.68	0.03	0.31	0.03	0.37	0.02
G80	34.67	0.83	39.33	0.73	59.83	0.88	65.84	0.43	17.99	0.35	33.89	0.32	12.80	0.35	4.44	0.46	11.08	0.75	0.75	0.04	0.35	0.03	0.40	0.03
G81	33.50	0.00	38.17	0.33	60.00	0.87	64.70	0.32	17.50	0.38	44.01	0.40	21.68	0.74	4.28	0.26	11.10	0.81	0.73	0.01	0.27	0.02	0.46	0.02
G82	34.00	0.50	38.67	0.17	60.67	1.09	74.84	0.32	16.88	0.31	43.83	0.44	18.87	0.68	4.45	0.59	12.81	0.76	0.86	0.06	0.19	0.05	0.66	0.03
G83	33.67	0.60	38.33	0.93	60.00	0.50	56.88	0.41	13.44	0.13	44.24	0.45	10.90	0.20	4.15	0.06	13.39	0.46	0.69	0.05	0.19	0.03	0.50	0.03
G84	33.50	0.58	38.17	0.88	64.83	0.33	44.95	0.38	15.19	0.11	43.97	0.39	9.50	0.47	4.13	0.08	11.66	0.33	1.37	0.07	0.76	0.06	0.61	0.02
G85	38.17	1.48	42.83	1.17	64.17	0.33	64.02	0.27	17.91	0.35	42.95	0.57	10.59	0.36	3.96	0.15	11.19	0.54	0.97	0.01	0.57	0.03	0.40	0.03
G86	38.67	0.73	43.33	0.44	61.00	0.50	65.20	0.33	17.56	0.28	44.37	0.36	9.64	0.35	4.17	0.17	12.34	0.63	0.84	0.05	0.34	0.04	0.51	0.02
G87	36.67	0.60	41.33	0.44	65.00	0.50	77.59	0.12	17.89	0.38	38.16	0.35	21.18	0.49	4.42	0.57	11.83	0.97	0.71	0.05	0.34	0.05	0.37	0.01
G88	36.33	1.42	41.00	1.32	60.33	0.17	59.49	0.30	18.35	0.40	34.21	0.39	9.99	0.42	3.78	0.15	11.74	0.66	0.84	0.05	0.40	0.03	0.45	0.02

G89	37.17	1.01	41.83	0.73	64.67	0.73	63.82	0.21	13.57	0.22	43.81	0.42	9.95	0.19	3.35	0.17	11.59	0.86	0.75	0.05	0.39	0.06	0.36	0.02
G90	39.67	1.42	44.33	1.09	64.83	0.73	52.60	0.35	18.03	0.37	33.34	0.13	9.95	0.19	3.98	0.20	11.86	0.46	0.98	0.01	0.50	0.03	0.48	0.02
G91	42.17	1.76	46.83	1.45	65.67	0.60	51.02	0.31	18.00	0.36	32.73	0.23	8.38	0.62	4.31	0.44	12.64	0.27	0.75	0.02	0.34	0.03	0.42	0.02
G92	46.50	1.16	51.17	0.88	65.67	1.01	60.09	0.24	17.44	0.65	32.85	0.30	8.37	0.15	4.25	0.17	11.86	0.46	0.83	0.05	0.45	0.07	0.39	0.02
G93	36.83	0.44	41.50	0.29	64.67	0.44	64.10	0.24	17.81	0.27	37.34	0.28	8.86	0.22	4.94	0.21	11.93	0.57	0.69	0.04	0.27	0.04	0.42	0.02
G94	40.17	0.73	44.83	1.01	66.67	0.17	41.74	0.22	13.57	0.21	48.23	0.42	9.50	0.47	4.74	0.40	10.00	0.97	0.67	0.04	0.33	0.04	0.34	0.01
G95	38.50	1.16	43.17	1.20	65.50	1.04	57.99	0.24	15.91	0.23	60.50	0.37	9.00	0.19	4.27	0.14	11.09	0.75	0.74	0.01	0.28	0.03	0.46	0.02
G96	34.50	0.00	39.17	0.33	63.00	0.76	64.89	0.33	14.47	0.68	26.71	0.18	9.95	0.19	4.03	0.32	12.14	0.82	0.76	0.06	0.26	0.05	0.51	0.01
G97	34.00	0.76	38.67	0.73	64.33	0.44	59.39	0.29	18.03	0.24	36.41	0.30	20.55	0.90	4.53	0.30	12.27	1.01	0.69	0.04	0.21	0.04	0.48	0.04
G98	37.67	1.59	42.33	1.42	61.67	0.33	68.17	0.09	17.79	0.20	38.27	0.29	16.33	0.38	4.03	0.56	12.37	0.64	0.74	0.02	0.23	0.04	0.52	0.04
G99	39.50	1.53	44.17	1.76	63.67	0.44	69.57	0.51	19.03	0.34	42.60	0.17	19.13	0.12	4.35	0.10	11.66	0.44	0.83	0.05	0.33	0.05	0.50	0.02
G100	32.50	1.53	37.17	1.33	63.33	0.33	53.95	0.14	18.91	0.34	42.78	0.36	12.03	0.30	3.60	0.56	9.34	0.94	0.99	0.02	0.46	0.05	0.53	0.03
G101	41.17	1.42	45.83	1.09	67.50	0.76	87.21	0.09	18.73	0.25	43.71	0.46	21.21	0.32	3.70	0.29	8.89	0.40	0.66	0.03	0.43	0.04	0.23	0.02
G102	38.67	1.69	43.33	1.36	67.83	0.60	67.91	0.18	19.10	0.29	48.56	0.30	11.08	0.29	3.70	0.26	10.68	1.31	0.86	0.02	0.51	0.01	0.35	0.02
G103	39.00	1.76	43.67	1.42	66.33	0.17	85.61	0.25	18.37	0.37	59.83	0.39	11.71	0.24	4.39	0.43	10.00	0.16	1.00	0.04	0.73	0.04	0.26	0.02
G104	46.00	0.76	50.67	0.73	69.33	0.60	68.54	0.51	18.81	0.28	26.83	0.28	13.47	0.11	4.60	0.28	12.20	0.49	0.85	0.02	0.24	0.01	0.62	0.02
G105	39.50	2.08	44.17	1.86	60.67	0.60	59.85	0.45	18.04	0.43	38.35	0.20	13.79	0.45	4.12	0.27	11.01	0.92	0.85	0.02	0.28	0.05	0.58	0.04
G106	42.17	0.33	46.83	0.33	68.50	0.58	68.34	0.40	16.66	0.38	38.67	0.27	18.95	0.08	5.50	0.47	11.52	0.92	0.86	0.02	0.41	0.01	0.45	0.02
G107	42.00	1.26	46.67	1.17	63.67	1.17	78.80	0.60	20.45	0.42	43.75	0.25	21.21	0.32	5.42	0.34	10.62	0.74	0.69	0.01	0.33	0.02	0.36	0.01
G108	40.50	0.76	45.17	0.44	63.33	0.60	70.23	0.18	18.10	0.23	37.54	0.16	13.12	0.42	4.51	0.57	12.03	0.44	0.72	0.05	0.28	0.05	0.45	0.02
G109	39.33	1.09	44.00	0.76	68.67	0.33	86.87	0.34	14.28	0.49	27.64	0.21	12.49	0.57	4.42	0.39	11.02	0.62	0.94	0.02	0.32	0.05	0.62	0.03
G110	38.17	1.17	42.83	0.83	59.33	0.93	86.47	0.19	13.25	0.48	43.31	0.07	23.89	0.18	4.39	0.05	12.38	0.93	0.87	0.02	0.45	0.05	0.42	0.04
G111	35.67	1.17	40.33	1.17	61.67	0.17	64.56	0.25	28.04	0.15	47.83	0.23	16.92	0.20	4.00	0.63	11.66	0.21	0.85	0.04	0.34	0.03	0.52	0.01
G112	46.33	1.48	51.00	1.26	80.33	1.01	74.71	0.47	28.77	0.20	33.14	0.32	11.22	0.17	4.48	0.10	10.30	0.77	0.66	0.02	0.34	0.03	0.33	0.01
G113	35.67	1.30	40.33	1.36	59.00	0.29	60.35	0.41	29.05	0.17	33.78	0.18	25.01	0.35	4.25	0.25	11.14	0.99	0.99	0.05	0.62	0.07	0.37	0.03
G114	34.67	0.44	39.33	0.17	65.33	0.17	52.67	0.28	28.65	0.17	33.89	0.14	11.40	0.49	5.18	0.17	11.39	0.40	0.68	0.03	0.42	0.04	0.26	0.01
G115	38.17	1.17	42.83	1.17	63.50	0.00	67.81	0.13	29.05	0.03	34.15	0.51	27.05	0.29	5.11	0.21	10.89	0.68	0.65	0.01	0.36	0.02	0.29	0.02
G116	34.67	1.48	39.33	1.17	61.17	0.44	46.71	0.28	28.71	0.36	33.66	0.56	30.68	0.55	3.64	0.06	10.27	0.96	0.68	0.03	0.43	0.04	0.24	0.01
G117	35.33	1.74	40.00	1.44	62.50	0.50	47.63	0.46	28.46	0.19	26.87	0.31	15.02	0.19	4.62	0.24	10.59	0.65	0.66	0.03	0.26	0.05	0.40	0.02
G118	35.50	0.76	40.17	0.44	64.17	0.44	58.52	0.31	28.69	0.24	44.04	0.40	25.47	0.26	3.46	0.14	11.36	0.48	0.69	0.04	0.37	0.04	0.31	0.03
G119	36.33	0.83	41.00	0.50	62.33	0.33	76.78	0.59	28.55	0.15	33.83	0.34	9.00	0.19	3.90	0.50	11.03	0.91	0.64	0.04	0.41	0.05	0.23	0.01
G120	34.83	1.30	39.50	1.04	66.33	0.44	52.79	0.25	29.78	0.20	33.74	0.38	22.49	0.88	4.02	0.33	11.49	0.34	0.66	0.02	0.43	0.03	0.23	0.01
G121	43.33	1.64	48.00	1.32	78.67	1.09	74.88	0.38	27.96	0.11	32.99	0.33	13.12	0.51	4.13	0.06	10.51	0.98	0.73	0.04	0.19	0.01	0.54	0.03
G122	35.17	0.73	39.83	0.44	65.00	0.50	53.02	0.25	28.76	0.10	42.06	0.11	14.24	0.07	3.76	0.27	11.53	0.48	0.87	0.02	0.63	0.02	0.24	0.02
G123	32.67	1.59	37.33	1.42	63.00	0.29	48.60	0.26	29.27	0.29	25.21	1.58	20.09	0.44	4.06	0.08	10.85	0.70	0.58	0.01	0.33	0.03	0.25	0.02
G124	39.00	0.76	43.67	1.09	66.83	0.33	52.44	0.30	28.87	0.32	38.63	0.28	26.91	0.37	3.78	0.37	12.43	0.67	0.60	0.00	0.31	0.02	0.29	0.02
G125	46.67	1.01	51.33	0.73	79.00	0.50	87.21	0.35	28.46	0.52	33.73	0.32	21.22	0.68	4.10	0.10	9.96	0.32	0.46	0.01	0.23	0.02	0.22	0.01
G126	33.83	0.33	38.50	0.00	61.33	0.33	47.83	0.17	28.60	0.48	27.81	0.24	36.09	0.17	5.62	0.39	12.20	0.62	0.83	0.02	0.26	0.04	0.57	0.03
G127	34.33	0.44	39.00	0.50	61.00	0.50	58.48	0.20	29.50	0.26	37.51	0.38	14.07	0.18	4.16	0.32	10.68	0.53	0.77	0.03	0.40	0.02	0.37	0.01
G128	35.33	1.59	40.00	1.61	64.00	0.29	53.52	0.22	25.31	0.30	37.58	0.41	25.47	0.26	4.16	0.47	12.37	0.65	0.97	0.05	0.40	0.05	0.57	0.02
G129	42.67	0.44	47.33	0.60	77.33	0.17	70.05	1.71	24.38	0.42	39.23	0.06	11.39	0.09	5.08	0.54	11.02	0.64	0.62	0.02	0.34	0.03	0.28	0.01
G130	34.00	0.29	38.67	0.17	62.33	0.73	53.94	0.58	28.70	0.25	34.28	0.12	21.53	0.37	4.28	0.28	10.68	0.32	0.60	0.01	0.38	0.02	0.22	0.01
G131	46.00	1.89	50.67	1.64	79.33	1.01	80.25	0.75	24.94	0.13	33.77	0.18	10.76	0.23	5.11	0.26	11.43	0.82	0.61	0.01	0.35	0.01	0.25	0.01
G132	37.33	1.42	42.00	1.26	62.67	1.30	73.44	0.29	25.10	0.24	33.69	0.42	12.98	0.31	5.39	0.15	11.01	0.86	0.74	0.02	0.16	0.01	0.58	0.03
G133	40.83	2.33	45.50	2.00	76.33	0.17	80.13	0.04	28.13	0.71	33.89	0.32	9.95	0.36	4.47	0.16	10.62	0.46	0.63	0.02	0.41	0.00	0.22	0.01
G134	37.17	2.19	41.83	2.33	64.33	0.93	60.37	0.36	23.50	0.25	48.30	0.28	5.52	0.15	3.71	0.28	12.54	0.92	0.75	0.01	0.25	0.02	0.49	0.02
G135	35.33	0.17	40.00	0.29	62.67	0.73	60.20	0.25	24.88	0.13	19.88	0.36	16.92	0.20	3.27	0.16	11.18	0.45	0.97	0.04	0.54	0.04	0.43	0.01
G136	35.33	1.36	40.00	1.50	65.17	0.44	69.25	0.33	24.59	0.34	42.74	0.68	9.95	0.19	4.38	0.40	11.81	0.78	0.62	0.01	0.22	0.02	0.40	0.03
G137	33.83	0.88	38.50	0.58	63.00	0.50	48.56	0.57	24.88	0.15	33.48	0.52	22.48	0.37	4.01	0.53	12.61	0.10	0.59	0.03	0.26	0.04	0.32	0.02
G138	41.83	2.33	46.50	2.65	82.00	0.29	59.85	0.17	25.55	0.64	32.99	0.31	9.95	0.19	4.71	0.18	9.80	0.79	0.46	0.01	0.26	0.01	0.20	0.01
G139	36.17	1.20	40.83	0.88	63.67	0.60	67.54	0.12	23.95	0.41	33.50	0.31	33.07	0.32	4.25	0.06	11.87	0.92	0.76	0.01	0.25	0.01	0.51	0.01
G140	36.00	1.32	40.67	1.01	61.33	0.83	52.90	0.08	28.66	0.29	33.71	0.47	19.00	0.56	5.50	0.43	12.51	0.61	0.51	0.01	0.21	0.01	0.30	0.01
G141	35.00	0.29	39.67	0.17	62.00	1.04	53.38	0.11	24.10	0.21	39.62	0.34	16.93	0.70	4.87	0.23	11.83	0.68	0.63	0.02	0.34	0.03	0.29	0.02
G142	36.83	1.45	41.50	1.16	63.67	1.09	53.14	0.24	28.65	0.30	33.91	0.31	20.26	0.37	4.69	0.25	12.03	0.91	0.64	0.02	0.35	0.02	0.28	0.02
G143	44.50	2.08	49.17	2.03	81.33	1.36	89.77	0.42	27.60	0.36	37.43	0.41	18.05	0.55	5.79	0.42	10.27	0.65	0.61	0.04	0.35	0.05	0.25	0.02
G144	46.33	0.60	51.00	0.87	82.67	0.60	87.51	0.26	28.22	0.36	33.46	0.33	16.93	0.70	4.40	0.12	10.10	0.35	0.47	0.04	0.24	0.03	0.23	0.01
G145	33.33	1.01	38.00	0.76	63.00	0.76	44.61	0.18	28.42	0.32	37.92	0.18	20.72	0.76	5.05	0.30	11.01	0.55	0.76	0.04	0.47	0.03	0.29	0.02
G146	37.33	1.97	42.00	1.76	61.50	0.76	44.21	0.35	28.67	0.28	43.65	0.29	7.29	0.75	4.45	0.57	11.35	0.46	0.76	0.01	0.23	0.01	0.52	0.02
G147	36.00	0.76	40.67	0.73	65.00	0.29	48.68	0.18	28.69	0.52	37.24	0.17	11.23	0.65	4.07	0.19	10.57	0.75	0.62	0.00	0.36	0.02	0.26	0.02
G148	34.67	0.17	39.33	0.44	69.67	0.17	63.82	0.23	24.38	0.33	43.35	0.44	14.25	0.51	4.44	0.36	10.85	0.24	0.46	0.03	0.24	0.02	0.23	0.01
G149	35.83	1.20	40.50	1.00	60.33	0.73	58.69	0.30	25.19	0.57	43.64	0.28	14.25	0.58	4.32	0.19	11.15	0.32	0.62	0.01	0.33	0.02	0.29	0.01
G150	36.00	1.50	40.67	1.17	61.83	0.93	53.15	0.41	25.83	0.27	34.22	0.29	18.68	0.35	4.63	0.40	12.44	0.88	0.63	0.01	0.30	0.02	0.33	0.02
G151	36.17	1.45	40.83	1.20	61.17	0.67	59.39	0.18	24.52	0.32	33.63	0.26	14.43	0.56	4.73	0.22	11.49	0.74	0.64	0.00	0.35	0.02	0.29	0.02
G152	33.83	0.88	38.50	0.58	66.50	0.50	61.25	0.21	24.25	0.49	47.67	0.17	11.53	0.47	3.48	0.27	8.87	0.81	0.50	0.01	0.30	0.03	0.21	0.03
G153	34.33	1.01	39.00	1.04	61.67	0.44	67.48	0.29	28.55	0.23	33.21	0.22	28.79	0.96	4.43	0.27	13.08	0.50	0.64	0.02	0.28	0.01	0.37	0.02
G154	44.00	0.76	48.67	0.44	80.67	1.48	72.09	0.23	28.51	0.51	32.00	0.43	16.80	0.93	4.82	0.37	10.82	0.47	0.63	0.01	0.36	0.03	0.27	0.02
G155	31.17	0.44	35.83	0.17	60.17	0.33	49.82	0.69	25.05	0.56	43.66	0.34	17.10	0.54	4.89	0.56	11.72	0.06	0.79	0.03	0.45	0.02	0.34	0.01
G156	34.83	1.09	39.50	0.76	74.67	1.17	58.56	1.89	22.53	0.53	21.05	0.51	18.37	0.83	4.67	0.26	12.44	0.56	0.50	0.01	0.20	0.00	0.30	0.01
G157	37.50	1.53	42.17	1.67	70.33	1.01	65.16	0.10	23.66	0.70	39.56	0.51	20.55	0.92	4.11	0.23	12.88	0.89	0.62	0.03	0.23	0.01	0.39	0.02
G158	35.17	0.93	39.83	0.60	66.67	0.73	65.63	0.49	24.40	0.46	53.43	0.64	20.09	0.44	4.68	0.26	12.41	0.42	0.61	0.02	0.29	0.02	0.32	0.01
G159	34.33	0.44	39.00	0.50	68.67	1.48	65.09	0.24	23.07	0.50	52.33	0.29	17.43	1.14	4.74	0.20	12.24	0.63	0.68	0.04	0.33	0.05	0.35	0.02
G160	33.33	1.17	38.00	0.87	63.50	0.58	54.64	0.21	23.99	0.47	50.02	0.34	18.91	1.19	5.14	0.25	10.23	1.18	0.59	0.02	0.27	0.01	0.32	0.01
G161	34.17	0.88	38.83	0.67	64.67	1.30	44.13	0.13	26.02	0.11	50.96	0.46	17.15	1.05	5.15	0.06	10.44	0.12	0.85	0.05	0.59	0.04	0.26	0.02
G162	33.33	0.83	38.00	0.50	65.33	1.36	56.46	0.25	27.29	0.46	47.93	0.49	16.83	0.79	4.43	0.55	12.17	0.68	0.62	0.02	0.28	0.02	0.34	0.01
G163	32.83	0.17	37.50	0.29	67.00	1.26	60.86	1.80	23.09	0.61	55.48	0.47	20.59	0.88	5.87	0.49	11.97	0.94	0.81	0.04	0.19	0.02	0.62	0.04
G164	33.33	0.60	38.00	0.29	67.67	1.88	63.00	0.61	25.08	0.89	54.74	0.88	21.85	0.61	5.52	0.38	9.66	0.84	0.61	0.02	0.27	0.02	0.34	0.02
G165	35.67	0.93	40.33	0.60	67.00	0.29	60.23	0.10	23.53	0.06	52.50	0.56	22.26	0.33	4.90	0.45	11.97	0.44	0.61	0.02	0.29	0.01	0.31	0.01
G166	35.00	0.50	39.67	0.17	72.67	0.44	58.22	0.41	26.05	0.27	43.16	0.40	21.68	0.74	5.25	0.44	11.42	0.33	0.56	0.03	0.22	0.01	0.34	0.02
**Level of Significance**	***		***		***		***		***		***		***		***		***		***		***		***	
**LSD**	**2.46**		**2.46**		**1.92**		**1.24**		**0.93**		**1.03**		**1.04**		**0.90**		**1.72**		**0.08**		**0.10**		**0.07**	
**SE(m)**	**0.88**		**0.88**		**0.69**		**0.45**		**0.33**		**0.37**		**0.37**		**0.32**		**0.62**		**0.03**		**0.04**		**0.02**	
**SE(d)**	**1.25**		**1.25**		**0.98**		**0.63**		**0.47**		**0.52**		**0.53**		**0.46**		**0.87**		**0.04**		**0.05**		**0.03**	
**C.V.**	**4.24**		**3.76**		**1.89**		**1.22**		**2.79**		**1.66**		**3.67**		**12.69**		**9.15**		**6.13**		**17.28**		**8.82**	

Results are represented as the mean value of triplicates ± standard error, SE = Standard error, LSD = least significant differences at 0.1% (***). DFF = days to first flowering, DF = days to flowering, DM = days to maturity, PH = plant height, CT = canopy temperature, SV = Spad value, NPPP = Number of pods per plant, NPPC = Number of pods per cluster, NSPP = Number of seeds per pod, PWt = pods weight, ShWtPP = shell weight per pod, SWtPP = seeds weight per pod

Table S6. Mean data of studied quantitative yield associated traits of mungbean genotypes in Season 2 (2019)

Genotypes	PL		TSW		YMV		YPP		StYPP		BY		HI	
	Mean	± S.E.	Mean	± S.E.	Mean	± S.E.	Mean	± S.E.	Mean	± S.E.	Mean	± S.E.	Mean	± S.E.
G1	6.32	0.17	21.76	0.62	1.68	0.23	6.18	0.38	29.59	0.96	35.27	0.59	17.41	1.38
G2	6.59	0.17	28.60	1.12	1.47	0.15	9.52	0.23	25.23	0.60	34.35	0.58	27.50	0.72
G3	6.74	0.18	31.64	0.50	1.31	0.11	16.70	0.35	23.08	0.74	39.42	0.60	42.05	1.00
G4	10.03	0.26	41.96	0.56	1.71	0.19	18.24	1.24	22.81	1.54	40.69	0.38	44.54	3.22
G5	7.57	0.20	47.26	1.03	2.11	0.11	14.27	1.82	24.95	1.58	38.83	0.28	36.38	4.29
G6	7.38	0.19	35.74	0.85	2.11	0.21	19.08	2.10	14.40	1.79	33.27	0.49	56.65	5.30
G7	10.55	0.28	48.33	1.16	1.26	0.07	22.34	1.84	17.66	1.51	39.73	0.72	55.70	3.94
G8	9.26	0.21	47.68	1.35	2.11	0.11	14.20	0.84	22.70	0.88	36.55	0.55	38.51	1.94
G9	8.24	0.22	41.08	1.13	2.66	0.31	16.76	0.96	13.17	1.17	29.73	0.44	55.99	3.50
G10	7.18	0.19	35.89	1.38	2.25	0.19	7.61	0.65	18.72	0.52	26.04	0.32	28.94	2.24
G11	7.86	0.21	47.90	1.65	2.27	0.28	9.51	0.84	27.40	0.66	36.49	0.51	25.80	1.90
G12	10.07	0.26	55.37	0.94	2.11	0.11	29.77	2.22	9.14	2.26	38.78	0.39	76.15	5.32
G13	8.11	0.23	48.63	1.36	1.73	0.30	14.83	0.56	15.61	0.40	30.19	0.16	48.74	1.55
G14	7.06	0.20	33.08	0.78	1.64	0.31	6.94	0.75	29.65	0.62	36.12	0.27	19.04	1.89
G15	7.63	0.22	32.06	0.69	2.96	0.23	4.62	0.37	33.89	0.20	37.97	0.18	12.07	0.91
G16	7.73	0.22	32.32	1.40	4.66	0.71	6.94	0.52	33.22	0.79	39.63	0.39	17.39	1.34
G17	8.66	0.21	47.87	1.24	2.34	0.22	15.72	0.14	23.22	0.57	38.57	0.60	40.53	0.47
G18	8.47	0.20	36.20	1.18	2.34	0.22	10.18	0.80	25.30	1.04	35.08	0.22	28.80	2.28
G19	7.48	0.16	50.76	1.33	2.11	0.11	12.38	0.96	28.56	1.13	40.49	0.45	30.23	2.28
G20	8.74	0.17	50.36	0.78	1.94	0.15	15.19	1.53	11.43	1.23	26.45	0.53	56.86	5.10
G21	8.24	0.16	41.08	1.09	1.58	0.25	6.70	0.65	31.88	0.61	38.08	0.29	17.45	1.68
G22	8.41	0.16	41.34	1.22	1.13	0.06	16.05	0.70	29.96	1.11	45.54	0.58	34.98	1.79
G23	8.41	0.16	41.19	1.10	1.30	0.20	9.72	0.34	31.62	0.08	40.84	0.28	23.57	0.72
G24	7.82	0.17	36.41	1.62	1.48	0.37	11.07	0.48	27.12	0.61	37.75	0.54	29.07	1.14
G25	7.13	0.16	22.59	0.99	2.11	0.11	5.96	0.42	15.97	0.60	21.68	0.44	27.32	1.84
G26	7.17	0.16	31.84	0.30	1.42	0.21	8.93	0.54	24.68	0.84	33.22	0.33	26.68	1.78
G27	8.58	0.16	41.24	0.97	1.38	0.10	12.04	0.75	20.00	1.04	31.72	0.49	37.73	2.52
G28	10.15	0.20	47.36	1.59	1.42	0.11	10.81	0.93	14.38	0.73	24.97	0.27	43.00	3.27
G29	8.81	0.16	47.93	1.44	1.99	0.23	15.48	0.26	14.46	0.24	29.71	0.50	51.67	0.16
G30	8.20	0.18	32.92	0.73	1.77	0.31	8.08	0.51	38.97	1.01	46.43	0.50	17.30	1.22
G31	7.58	0.15	40.51	0.61	1.66	0.12	8.85	0.39	28.81	0.33	37.20	0.28	23.60	0.85
G32	9.34	0.18	47.90	1.43	1.76	0.23	7.84	0.18	31.67	0.32	39.01	0.39	19.96	0.41
G33	7.01	0.15	32.92	0.60	3.91	0.17	6.31	0.42	27.82	0.83	33.69	0.96	18.41	1.08
G34	8.27	0.15	41.16	1.18	3.20	0.72	6.67	0.30	33.57	0.45	39.71	0.40	16.62	0.63
G35	9.73	0.19	56.06	1.79	2.60	0.06	11.43	0.52	15.59	0.17	26.78	0.35	42.19	1.36
G36	7.44	0.16	31.54	0.94	2.34	0.22	7.29	0.45	18.69	0.28	25.69	0.64	27.95	1.18
G37	8.63	0.17	46.52	1.76	2.79	0.54	12.19	0.61	28.72	0.20	40.46	0.53	29.82	1.13
G38	8.84	0.18	47.04	1.91	1.36	0.17	16.24	1.04	10.56	1.66	26.65	0.60	60.49	5.08
G39	8.03	0.17	47.08	0.82	2.25	0.19	8.68	0.86	29.67	1.24	37.88	0.51	22.73	2.38
G40	6.62	0.14	31.10	0.42	2.11	0.11	7.37	0.57	26.01	0.94	32.96	0.39	22.11	1.87
G41	8.11	0.17	36.57	1.17	1.36	0.17	4.55	0.14	35.41	0.66	39.40	0.53	11.48	0.52
G42	7.87	0.16	54.94	0.50	2.44	0.11	9.22	0.66	27.52	0.20	36.30	0.53	25.27	1.38
G43	8.79	0.18	31.52	0.51	4.66	0.11	13.42	0.66	22.31	0.76	35.38	0.46	37.65	1.85
G44	8.34	0.17	50.23	0.50	1.94	0.15	15.66	1.52	22.84	1.10	38.14	0.45	40.62	3.21
G45	7.90	0.17	36.14	1.42	3.45	0.11	14.15	0.56	27.40	0.34	41.12	0.29	34.06	1.00
G46	9.17	0.19	44.52	0.71	3.92	0.20	26.64	0.83	11.21	0.67	37.69	0.35	70.18	1.65
G47	7.95	0.17	41.26	1.03	2.64	0.41	12.81	0.85	26.10	0.51	38.50	0.55	33.01	1.82
G48	9.98	0.21	46.51	1.39	5.15	0.30	10.13	0.19	31.58	0.41	41.21	0.43	24.42	0.35
G49	8.34	0.17	49.72	0.81	2.31	0.51	21.47	1.07	16.74	0.90	37.96	0.53	56.02	2.08
G50	9.46	0.19	46.97	1.53	1.94	0.15	12.28	0.32	29.65	0.64	41.47	0.51	29.36	0.81
G51	8.58	0.18	31.00	0.76	3.13	0.11	5.00	0.60	18.84	0.36	23.54	0.50	21.00	2.14
G52	8.69	0.18	32.00	1.08	1.94	0.15	9.02	0.19	18.92	0.50	27.64	0.37	32.37	0.80
G53	7.14	0.15	35.50	0.97	5.55	0.15	4.84	0.45	35.09	0.13	39.37	0.46	12.19	1.01
G54	7.26	0.16	32.59	1.41	5.85	0.25	3.48	0.15	37.57	0.27	40.46	0.42	8.53	0.26
G55	7.47	0.16	44.54	0.57	5.85	0.25	15.06	0.96	23.48	0.33	38.17	0.84	39.12	1.49
G56	7.56	0.16	41.18	0.60	2.79	0.06	24.82	0.62	13.86	0.07	38.46	0.55	64.25	0.50
G57	7.02	0.15	55.18	0.91	4.70	0.30	6.63	0.80	23.16	0.78	29.43	0.51	22.33	2.56
G58	7.23	0.16	45.68	1.83	4.26	0.19	4.70	0.39	33.13	0.72	37.29	1.06	12.50	0.83
G59	7.68	0.19	41.45	1.70	3.41	0.25	12.04	1.19	26.09	1.44	37.73	0.46	31.64	3.20
G60	8.43	0.20	56.06	1.20	4.26	0.40	8.61	0.99	31.34	1.34	39.45	0.52	21.71	2.56
G61	8.91	0.21	47.33	1.29	4.09	0.26	10.91	0.38	16.75	0.90	27.40	0.54	39.63	1.88
G62	7.72	0.19	37.24	1.20	4.95	0.19	4.51	0.53	29.24	0.33	33.28	0.51	13.43	1.43
G63	9.18	0.22	46.80	1.35	5.12	0.43	3.04	0.47	37.40	0.73	39.85	0.25	7.62	1.25
G64	7.37	0.18	32.24	0.42	4.94	0.20	4.11	0.48	29.73	0.50	33.36	0.55	12.27	1.30
G65	9.46	0.22	50.41	0.17	4.32	0.30	8.33	0.36	29.97	0.53	37.82	0.57	21.85	0.87
G66	8.95	0.21	41.05	1.10	1.36	0.17	11.63	1.10	29.82	1.33	40.98	0.26	28.15	2.73
G67	8.55	0.20	46.81	1.61	1.13	0.06	12.89	0.48	26.59	0.41	39.06	0.29	32.73	1.14
G68	6.77	0.17	31.45	0.44	1.48	0.37	5.51	0.36	32.83	0.30	37.82	0.57	14.49	0.71
G69	6.85	0.18	22.36	1.01	1.76	0.23	8.66	0.61	15.38	1.07	23.80	0.74	36.21	2.93
G70	7.44	0.19	56.44	1.57	2.11	0.21	8.94	0.47	32.19	0.33	40.61	0.58	21.85	0.80
G71	9.04	0.20	41.27	0.66	1.13	0.06	7.80	0.27	23.29	0.50	30.72	0.32	25.27	1.02
G72	7.55	0.18	40.95	0.68	1.71	0.11	10.64	0.92	27.06	1.30	37.26	0.46	28.45	2.71
G73	10.13	0.24	41.40	0.69	1.36	0.17	5.56	0.28	30.52	0.92	35.59	0.62	15.55	1.01
G74	7.49	0.18	31.23	0.25	1.26	0.07	7.00	0.47	28.53	0.48	35.08	0.43	19.72	1.20
G75	8.39	0.19	36.35	1.01	2.39	0.10	5.34	0.31	32.05	0.82	36.88	0.55	14.40	1.00
G76	6.74	0.16	32.19	0.71	1.13	0.06	4.35	0.06	25.18	0.81	29.13	0.74	14.82	0.61
G77	8.46	0.19	41.27	0.66	1.13	0.06	7.98	0.15	20.96	0.35	28.61	0.45	27.65	0.26
G78	7.97	0.19	44.92	0.38	1.48	0.15	11.84	0.46	26.79	0.09	38.21	0.44	30.72	0.84
G79	6.86	0.16	31.00	0.48	1.77	0.24	10.55	0.76	29.63	1.02	39.71	0.36	26.43	1.97
G80	7.44	0.17	31.29	0.19	1.94	0.38	5.30	0.54	28.19	0.86	33.04	0.31	15.94	1.75
G81	8.08	0.17	35.71	0.73	1.13	0.06	10.57	0.66	13.11	0.99	23.47	0.61	44.80	2.84
G82	9.41	0.19	45.37	1.44	1.94	0.15	13.00	0.63	14.81	0.56	27.57	0.57	46.89	1.77
G83	8.55	0.17	40.73	0.38	2.06	0.26	5.60	0.51	35.47	0.69	40.51	0.45	13.73	1.25
G84	7.25	0.16	50.62	0.60	1.13	0.06	5.87	0.37	31.89	1.06	37.25	0.68	15.70	1.30
G85	7.40	0.16	32.09	0.61	1.60	0.31	4.29	0.32	33.48	1.26	37.24	0.92	11.54	1.13
G86	7.60	0.16	36.44	0.95	1.50	0.12	4.91	0.13	30.62	0.49	35.04	0.42	13.95	0.51
G87	7.01	0.15	28.35	1.49	1.13	0.06	8.52	0.39	30.53	0.62	38.57	0.23	21.94	1.10
G88	7.23	0.16	31.69	0.27	1.48	0.37	4.61	0.39	34.99	1.03	39.04	0.92	11.73	0.98
G89	8.59	0.18	28.03	1.37	1.92	0.40	3.71	0.41	33.83	0.50	37.00	0.90	9.92	0.87
G90	8.84	0.18	36.20	0.39	2.35	0.10	4.82	0.43	35.51	0.66	39.76	0.23	12.01	1.14
G91	8.02	0.18	28.70	1.38	1.75	0.23	3.65	0.41	37.16	0.69	40.22	0.30	9.01	1.08

G92	6.50	0.13	30.55	0.84	1.13	0.06	3.29	0.39	21.55	0.16	24.50	0.53	13.25	1.31
G93	8.08	0.17	31.82	0.99	1.77	0.24	3.74	0.11	33.12	1.00	36.34	1.06	10.21	0.20
G94	8.46	0.17	28.48	0.94	1.48	0.37	3.31	0.14	23.53	0.55	26.46	0.62	12.43	0.47
G95	7.92	0.17	36.61	1.01	1.13	0.06	4.20	0.27	23.96	0.61	27.78	0.57	15.05	0.98
G96	7.07	0.16	36.88	1.26	1.73	0.27	4.96	0.36	35.10	0.80	39.49	0.50	12.52	1.03
G97	6.89	0.15	36.65	1.39	1.13	0.06	10.57	0.58	26.23	1.64	36.39	1.03	28.89	2.29
G98	6.04	0.14	40.03	0.35	1.13	0.06	8.78	0.65	32.06	0.84	40.33	0.50	21.62	1.54
G99	5.86	0.14	41.36	0.78	1.77	0.24	9.79	0.69	14.79	0.54	24.35	0.45	39.86	2.44
G100	8.34	0.17	44.51	1.45	1.47	0.38	6.58	0.60	29.24	0.52	35.35	0.43	18.45	1.58
G101	7.85	0.15	27.29	0.90	1.65	0.35	5.00	0.68	28.85	0.25	33.40	0.58	14.77	1.73
G102	7.78	0.17	35.34	0.79	2.62	0.31	4.00	0.36	35.97	0.73	39.40	0.54	10.10	0.94
G103	8.53	0.17	27.68	1.09	2.22	0.40	3.13	0.34	37.99	0.84	40.52	0.53	7.71	0.91
G104	8.98	0.17	51.19	1.13	1.47	0.38	8.52	0.60	30.12	0.42	38.16	0.20	22.13	1.46
G105	7.93	0.16	40.86	0.74	1.60	0.31	8.38	0.76	28.89	0.62	36.81	0.28	22.58	1.89
G106	7.06	0.15	35.25	1.04	1.47	0.38	8.70	0.49	28.27	0.19	36.53	0.32	23.64	1.11
G107	6.96	0.15	35.68	1.52	1.48	0.37	7.93	0.56	25.08	0.47	32.62	0.32	24.05	1.53
G108	8.01	0.18	35.61	0.87	1.13	0.06	6.12	0.29	24.20	0.24	29.94	0.35	20.23	0.74
G109	8.92	0.18	48.09	1.02	1.13	0.06	8.10	0.42	28.94	0.65	36.58	0.57	21.98	1.06
G110	8.36	0.17	31.84	0.94	1.13	0.06	10.51	0.82	30.51	1.33	40.53	0.48	25.74	2.12
G111	7.70	0.16	41.00	0.91	1.81	0.21	8.91	0.27	38.94	0.18	47.24	0.42	18.73	0.45
G112	6.03	0.14	27.20	1.18	1.43	0.15	3.72	0.25	21.97	0.60	25.34	0.46	14.61	1.14
G113	7.14	0.16	31.03	0.72	1.13	0.06	9.73	0.93	17.11	0.56	26.58	0.40	36.21	2.78
G114	5.70	0.13	22.84	0.97	1.98	0.23	3.00	0.06	26.02	0.61	28.62	0.58	10.41	0.32
G115	6.44	0.14	23.45	1.16	1.26	0.07	8.17	0.57	18.60	1.02	26.47	0.49	30.60	2.35
G116	6.11	0.14	22.41	0.69	1.13	0.06	7.85	0.37	24.51	0.39	31.98	0.31	24.20	0.96
G117	5.45	0.13	30.92	0.84	3.13	0.21	6.18	0.56	40.00	0.50	45.54	0.49	13.44	1.15
G118	6.28	0.14	24.38	1.68	1.13	0.06	8.42	0.70	17.57	1.28	25.71	0.56	32.47	3.27
G119	5.73	0.09	22.08	1.33	1.94	0.15	1.98	0.10	22.78	0.21	24.40	0.25	8.05	0.35
G120	6.12	0.14	18.43	1.06	1.89	0.19	5.53	0.41	23.04	0.38	28.21	0.27	19.44	1.37
G121	5.45	0.09	45.47	1.06	1.13	0.06	7.13	0.65	14.98	0.30	21.88	0.36	32.34	2.34
G122	5.08	0.09	18.39	1.05	1.43	0.15	3.57	0.26	20.18	0.87	23.42	0.61	15.19	1.43
G123	5.56	0.09	22.73	1.33	1.13	0.06	5.18	0.28	20.06	0.50	24.92	0.29	20.60	1.12
G124	6.75	0.10	22.25	0.90	1.54	0.21	8.19	0.25	17.96	0.10	25.87	0.34	31.35	0.55
G125	5.10	0.09	22.94	1.16	2.45	0.15	4.90	0.14	21.79	0.37	26.34	0.44	18.54	0.41
G126	7.62	0.11	40.70	1.06	2.41	0.36	21.51	1.37	11.36	1.03	32.70	0.55	65.18	3.22
G127	7.58	0.11	36.52	1.09	1.94	0.15	5.27	0.20	28.75	0.69	33.57	0.54	15.60	0.78
G128	8.26	0.11	46.25	1.19	2.11	0.11	15.40	0.98	22.93	0.63	37.98	0.36	40.11	2.08
G129	6.74	0.10	21.74	0.87	1.13	0.06	3.21	0.22	23.25	0.53	26.09	0.45	12.23	0.89
G130	6.50	0.10	17.81	1.35	1.81	0.21	4.96	0.33	23.93	0.46	28.51	0.29	17.35	1.06
G131	4.58	0.08	22.39	0.89	1.60	0.10	2.85	0.15	25.46	0.93	27.91	0.90	10.12	0.61
G132	4.87	0.08	54.86	0.68	2.33	0.11	7.70	0.53	25.31	0.55	32.61	0.63	23.36	1.40
G133	5.16	0.09	22.14	1.18	2.62	0.39	2.13	0.07	34.09	0.50	35.68	0.42	5.92	0.23
G134	7.10	0.10	30.09	0.91	1.13	0.06	2.58	0.28	38.60	0.67	40.55	0.42	6.42	0.78
G135	9.66	0.12	34.61	1.07	3.64	0.46	7.44	0.30	34.61	0.80	41.50	0.55	17.76	0.92
G136	4.97	0.09	31.99	1.12	2.62	0.32	4.03	0.19	31.97	0.65	35.49	0.50	11.28	0.59
G137	6.15	0.09	22.47	0.88	1.26	0.07	7.72	0.34	31.26	0.36	38.48	0.26	19.91	0.79
G138	4.85	0.08	22.86	0.98	2.11	0.11	1.99	0.13	38.81	0.42	40.18	0.29	4.90	0.36
G139	7.38	0.12	40.60	0.85	1.26	0.07	17.72	0.45	30.50	0.68	47.74	0.55	36.88	0.88
G140	6.18	0.13	23.18	1.09	2.11	0.11	5.96	0.19	31.90	0.53	37.36	0.41	15.83	0.55
G141	6.07	0.09	24.00	2.12	1.13	0.06	5.26	0.23	19.99	0.36	24.94	0.29	20.92	0.88
G142	7.01	0.11	21.58	1.02	1.54	0.21	6.05	0.70	20.97	1.19	26.70	0.59	22.54	2.78
G143	5.44	0.08	22.84	0.77	1.60	0.31	4.83	0.51	26.35	0.64	30.75	0.29	15.56	1.62
G144	6.73	0.11	22.29	0.98	1.60	0.31	4.12	0.23	26.18	0.14	29.89	0.37	13.63	0.60
G145	6.84	0.11	23.60	1.03	1.60	0.32	6.39	0.49	30.44	0.71	36.35	0.39	17.41	1.32
G146	6.60	0.11	41.22	0.28	2.90	0.19	4.09	0.39	30.96	0.36	34.57	0.56	11.71	1.02
G147	5.66	0.08	23.34	1.18	4.15	0.21	3.16	0.34	36.43	0.05	39.01	0.33	8.02	0.78
G148	4.53	0.08	21.80	1.25	4.69	0.18	3.44	0.23	32.62	0.46	35.54	0.47	9.58	0.61
G149	6.55	0.11	23.78	1.28	2.96	0.37	4.40	0.28	18.06	0.64	22.17	0.36	19.82	1.57
G150	6.30	0.10	23.07	0.98	3.64	0.40	6.46	0.26	17.15	0.47	23.34	0.25	27.42	1.20
G151	6.02	0.11	23.48	1.53	3.64	0.11	4.41	0.20	19.00	0.39	23.10	0.39	18.93	0.82
G152	5.60	0.09	24.46	0.93	1.43	0.14	2.61	0.56	17.38	0.23	19.72	0.33	13.05	2.54
G153	6.54	0.09	27.66	0.68	1.48	0.23	11.33	0.78	24.81	0.82	35.75	0.54	31.40	2.00
G154	5.37	0.08	23.73	1.77	1.13	0.06	4.65	0.57	20.08	0.28	24.41	0.42	18.85	2.07
G155	7.69	0.12	25.97	0.86	3.13	0.11	6.23	0.47	22.27	0.35	28.15	0.30	21.96	1.54
G156	8.85	0.11	21.53	0.35	4.60	0.39	5.82	0.32	26.38	0.28	31.79	0.37	18.17	0.88
G157	8.42	0.11	27.96	1.10	4.49	0.41	8.58	0.61	28.22	0.97	36.36	0.64	23.42	1.70
G158	8.36	0.11	23.48	0.46	3.47	0.40	6.83	0.46	30.25	0.83	36.60	0.64	18.53	1.30
G159	8.22	0.11	25.85	0.08	2.67	0.57	6.38	0.16	33.88	0.65	39.72	0.54	15.96	0.56
G160	7.78	0.12	25.48	0.13	1.13	0.06	6.22	0.36	32.41	0.41	38.11	0.31	16.20	0.92
G161	8.09	0.11	21.24	0.31	1.43	0.24	4.65	0.53	35.96	0.64	40.04	0.29	11.52	1.31
G162	8.09	0.11	26.78	0.35	1.13	0.06	5.93	0.24	35.25	0.73	40.61	0.49	14.50	0.77
G163	8.86	0.13	45.75	0.12	1.82	0.40	13.47	1.03	24.70	1.61	37.78	0.54	35.48	3.11
G164	7.85	0.11	24.76	0.30	3.13	0.60	7.85	0.52	33.29	0.94	40.62	0.44	19.20	1.45
G165	7.72	0.10	23.32	0.45	2.11	0.11	7.29	0.39	30.93	0.93	37.72	0.61	19.20	1.25
G166	7.32	0.10	26.96	0.53	3.47	0.23	7.72	0.56	26.68	0.42	33.98	0.38	22.54	1.50
**Level of Significance**	***		***		***		***		***		***		***	
**LSD**	**0.18**		**2.67**		**0.67**		**1.53**		**2.03**		**1.33**		**4.31**	
**SE(m)**	**0.07**		**0.96**		**0.24**		**0.55**		**0.73**		**0.48**		**1.55**	
**SE(d)**	**0.09**		**1.36**		**0.34**		**0.78**		**1.03**		**0.67**		**2.19**	
**C.V.**	**1.49**		**4.69**		**18.93**		**11.19**		**4.81**		**2.40**		**10.81**	

Results are represented as the mean value of triplicates ± standard error, SE = Standard error, LSD = least significant differences at 0.1% (***). PL = pod length, TSW = 1000 seed weight, YMV = yellow mosaic virus, YPP = yield per plant (g), SYPP = stover yield per plant, BYPP = biological yield per plant(g), and HI = harvest index

Table S7. Eigenvalue (latent roots), contribution to variability, and factor loading for the principal components (PCs) analysis of the quantitative traits in 166 mungbean genotypes

Quantitative traits	PC1	PC2	PC3	PC4	PC5	PC6	PC7	PC8	PC9	PC10
DFF	0.564	**-0.217**	**0.651**	-0.046	-0.071	0.014	-0.079	0.032	0.037	0.129
DF	0.589	**-0.247**	**0.700**	-0.066	-0.091	0.024	-0.099	0.052	0.047	0.149
DM	**0.712**	**-0.143**	0.385	0.066	-0.072	0.021	0.042	0.034	0.176	0.126
PH	0.097	-0.171	**0.599**	0.012	-0.072	-0.187	0.191	**-0.536**	-0.276	-0.303
CT	**0.431**	**-0.451**	-0.283	0.153	-0.332	0.094	-0.026	-0.015	0.334	0.194
SV	0.01	0.254	-0.083	-0.011	-0.187	**-0.839**	**0.322**	0.066	0.033	0.258
PPP	-0.392	**-0.734**	-0.211	**0.180**	-0.361	-0.034	0.008	-0.151	0.04	-0.075
CPP	0.035	-0.344	0.101	**0.506**	-0.119	0.083	0.185	0.502	**-0.538**	0.032
SPP	**-0.547**	0.030	-0.002	0.122	0.091	0.008	-0.518	-0.296	-0.283	**0.450**
SPWt	**-0.783**	0.249	**0.273**	-0.344	-0.233	0.164	0.09	0.114	0.017	0.062
ShWtPP	-0.187	0.233	-0.047	**-0.623**	-0.6	**0.280**	0.206	-0.002	-0.162	0.088
SWtPP	**-0.844**	0.126	**0.386**	0.071	0.194	-0.019	-0.054	0.147	0.153	0.008
PL	**-0.741**	**0.217**	0.193	-0.03	-0.009	-0.081	0.042	-0.038	-0.066	0.181
TSW	**-0.769**	0.126	**0.451**	0.033	0.185	-0.013	0.058	0.195	0.231	-0.113
YMV	-0.059	0.216	0.016	0.405	0.237	0.381	**0.636**	**-0.279**	0.083	0.28
YPP	**-0.786**	-0.519	0.073	**0.178**	-0.195	-0.029	0.005	-0.035	0.115	-0.021
SYPP	0.279	**0.818**	0.066	0.362	-0.291	0.038	-0.141	-0.021	0.026	-0.085
BYPP	-0.32	0.511	0.135	**0.558**	**-0.490**	0.02	-0.157	-0.053	0.123	-0.114
HI	**-0.723**	-0.670	0.024	-0.003	**-0.016**	-0.028	0.057	-0.016	**0.069**	0.001
**Eigenvalues**	5.676	2.989	2.122	1.492	1.231	1.018	0.975	0.817	0.744	0.622
**Proportion% Variance**	29.9	15.7	11.2	7.9	6.5	5.4	5.1	4.3	3.9	3.3
**Cumulative Variance**	29.9	45.6	56.8	64.6	71.1	76.5	81.6	85.9	89.8	93.1

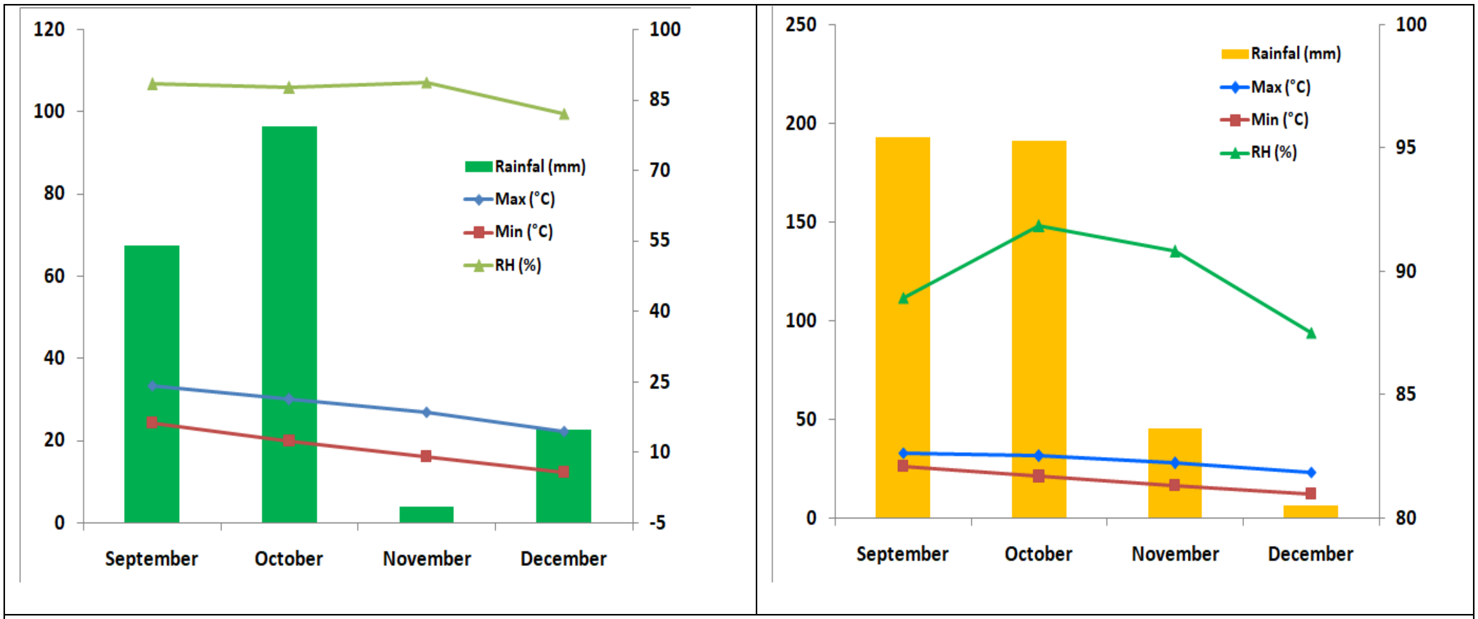

Figure S1. Meteorological data at the experimental site at PRC, BARI for two consecutive trials

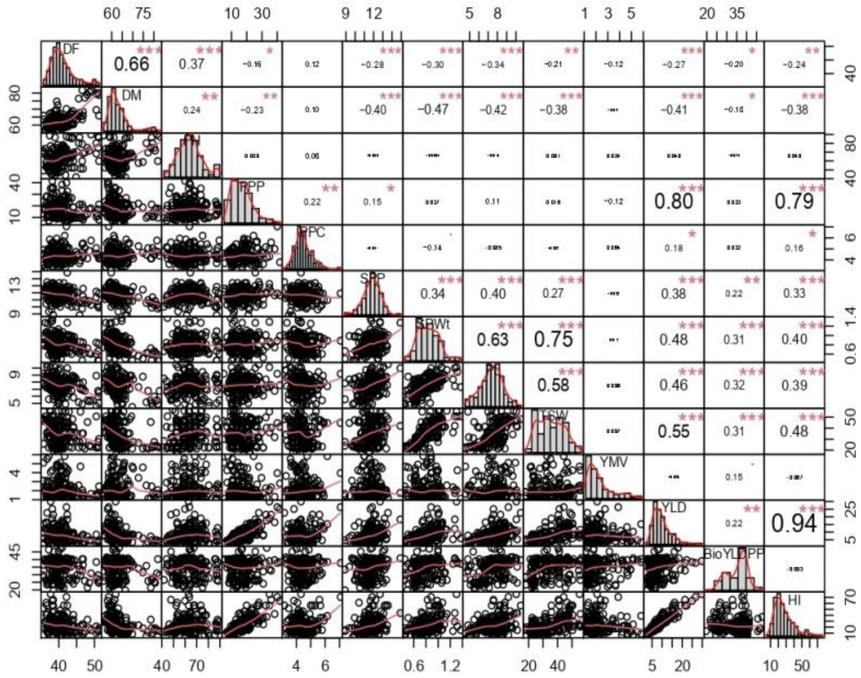

Figure S2. The pair-wise correlations of different 13 quantitative traits of 166 mungbean germplasm lines. The scatter plots (below diagonal), and values of the correlation (above diagonal) between pairs of traits are shown. * Significant at the 5% global level ($p < 0.05$), ** Significant at the 1% global level ($p < 0.01$), *** Significant at the 0.1% global level ($p < 0.001$). Abbreviations: DF = days to flowering, DM = days to maturity, PH = plant height, PPP = Number of pods per plant, PPC = Number of pods per cluster, SPP = Number of seeds per pod, PWt = single pods weight, PL = pod length, TSW = 1000 seed weight, YMV = yellow mosaic virus, YPP = yield per plant, BYPP = biological yield per plant, and HI = harvest index used as experimental attributes, respectively.

Table S8. Distribution of 166 mungbean germplasm in each cluster connecting agronomic characters based on their Euclidean distance, following the ward's method.

**Clusters**	**Number of genotypes**	**Name of Genotype code included**
Cluster I	20	G1 G2 G117 G136 G137 G140 G145 G147, G148 G153 G156 G157 G158 G159 G160 G161 G162 G164 G165 and G166
Cluster II	34	G3 G4 G5 G6 G7 G8 G9 G12 G13 G17 G19 G20 G22 G23 G24 G27 G28 G29 G35, G38 G43 G44 G45 G46 G49 G55 G56 G59 G61 G71 G82, G126 G128 and G139
Cluster III	28	G10 G18 G25 G26 G31 G36 G40 G51 G52 G68 G69 G72 G74 G76 G77 G79 G80 G81 G87 G93 G96 G97 G98 G99 G105 G108 G110 and G113
Cluster IV	33	G11 G16 G21 G30 G32 G33 G34 G37 G39 G42 G48 G50 G53 G54 G57 G58 G60 G62 G63 G64 G65 G66 G67 G70 G73 G75 G78 G83 G84 G100 G132 G135 and G163
Cluster V	17	G114 G115 G116 G118 G119 G120 G122 G123 G124 G130 G141 G142 G149 G150, G151 G152 and G155
Cluster VI	24	G14 G15 G41 G47 G85 G86 G88 G89 G90 G91 G92 G94 G95 G101 G102 G103 G104 G106 G107 G109 G111 G127 G134 and G146
Cluster VII	10	G112 G121 G125 G129 G131 G133 G138 G143 G144 and G154
**Total**	**166**	
